# Association of cardiac myosin-binding protein-C with the ryanodine receptor channel – putative retrograde regulation?

**DOI:** 10.1242/jcs.210443

**Published:** 2018-08-03

**Authors:** Paulina J. Stanczyk, Monika Seidel, Judith White, Cedric Viero, Christopher H. George, Spyros Zissimopoulos, F. Anthony Lai

**Affiliations:** 1 Sir Geraint Evans Wales Heart Research Institute, Department of Cardiology, School of Medicine, Cardiff University, Cardiff CF14 4XN, UK; 2 School of Biosciences, Sir Martin Evans Building, College of Biomedical and Life Sciences, Cardiff University, Cardiff CF10 3AX, UK; 3 Swansea University Medical School, Institute of Life Science, Swansea SA2 8PP, UK; 4 Institute of Pharmacology and Toxicology, Medical School, Saarland University, Homburg/Saar, Germany; 5 College of Medicine, Member of QU Health, Qatar University, P.O. Box 2013, Doha, Qatar

**Keywords:** Ca^2+^ signalling, Myosin-binding protein C, Sarcomere, Ryanodine receptor, Sarcoplasmic reticulum, Excitation–contraction coupling

## Abstract

The cardiac muscle ryanodine receptor-Ca^2+^ release channel (RyR2) constitutes the sarcoplasmic reticulum (SR) Ca^2+^ efflux mechanism that initiates myocyte contraction, while cardiac myosin-binding protein-C (cMyBP-C; also known as MYBPC3) mediates regulation of acto-myosin cross-bridge cycling. In this paper, we provide the first evidence for the presence of direct interaction between these two proteins, forming a RyR2–cMyBP-C complex. The C-terminus of cMyBP-C binds with the RyR2 N-terminus in mammalian cells and the interaction is not mediated by a fibronectin-like domain. Notably, we detected complex formation between both recombinant cMyBP-C and RyR2, as well as between the native proteins in cardiac tissue. Cellular Ca^2+^ dynamics in HEK293 cells is altered upon co-expression of cMyBP-C and RyR2, with lowered frequency of RyR2-mediated spontaneous Ca^2+^ oscillations, suggesting that cMyBP-C exerts a potential inhibitory effect on RyR2-dependent Ca^2+^ release. Discovery of a functional RyR2 association with cMyBP-C provides direct evidence for a putative mechanistic link between cytosolic soluble cMyBP-C and SR-mediated Ca^2+^ release, via RyR2. Importantly, this interaction may have clinical relevance to the observed cMyBP-C and RyR2 dysfunction in cardiac pathologies, such as hypertrophic cardiomyopathy.

## Abstract (precis)

**Highlighted Article:** Myosin binding protein-C functionally interacts with the cardiac Ca^2+^ release channel (RyR2), suggesting a novel retrograde regulation mechanism for muscle excitation–contraction coupling.

## INTRODUCTION

Cardiac muscle excitation–contraction coupling, where the electrical depolarisation stimulus of cardiomyocytes plasma membrane triggers mechanical contraction of the heart, is mediated by Ca^2+^-induced Ca^2+^-release, where a small influx of Ca^2+^ into the cytoplasm results in release of Ca^2+^ from SR. When the heart is in the diastolic (relaxation) phase, intracellular Ca^2+^ is sequestered into the sarcoplasmic reticulum (SR), the major internal Ca^2+^ store of the cardiomyocyte. During systole (the contraction phase), plasma membrane electrical excitation causes Ca^2+^ influx through coordinated opening of voltage-gated Ca^2+^ channels, leading to a global efflux of Ca^2+^ from the SR via the SR-located Ca^2+^ release channel-ryanodine receptor 2 complex (RyR2), which in turn induces sarcomere shortening (Bers, 2002). Physiological regulation of muscle contraction involves the control of acto-myosin cross-bridge cycling, which is initiated by Ca^2+^ binding to troponin, thus allowing the myosin thick-filament protein to interact with actin, the thin-filament protein. The force and frequency of muscle contraction is influenced by numerous factors including sarcomere Ca^2+^ responsiveness, phosphorylation and various modulatory proteins; among the latter is myosin-binding protein C (MyBP-C), an important protein that plays a vital modulatory role (Clark et al., 2002; Moss et al., 2015).

The three mammalian RyR protein isoforms (skeletal RyR1, cardiac RyR2 and RyR3, which is expressed in various tissues) share ∼65% sequence identity, with the concensus RyR oligomeric structure composed of four identical subunits surrounding a centrally located Ca^2+^ ion channel pore entailing the C-terminal transmembrane domains of each protomer. In contrast, the much larger cytoplasmic N-terminal assembly serves as a binding scaffold for myriad accessory proteins, ions and other regulatory molecules (Lanner et al., 2010). Modulation of RyR channel opening and closing by stimulatory/inhibitory cytoplasmic signals is mediated by long-range conformational changes involving the interplay between N- and C-terminal domains within a single RyR subunit, as well as between neighbouring subunits of the RyR tetramer (Seidel et al., 2015a). Given the essential role of RyR in enabling SR Ca^2+^ release, it is unsurprising that functional alterations of RyRs are associated with human disease (Lanner et al., 2010). Thus, RyR1 mutations are linked to skeletal muscle disorders including malignant hyperthermia and central core disease, while RyR2 mutations have a direct causative role in the heart rhythm disorders catecholaminergic polymorphic ventricular tachycardia (CPVT) and arrhythmogenic right ventricular dysplasia (ARVD), and discrete RyR2 mutations have also been recently implicated in dilated cardiomyopathy (DCM) (Haas et al., 2014).

MyBP-C is an immunoglobulin (Ig) protein superfamily member and exists in three isoforms: cardiac [cMyBP-C, which is exclusively heart expressed (also known as MYBPC3)], and fast- and slow-skeletal muscle types (also known as MYBPC2 and MYBPC1, respectively) with an overall peptide sequence identity of ∼55%. All three isoforms share common architectural features, comprising ten globular domains designated C1–C10, seven of which are Ig-like domains with the remaining three categorised as fibronectin-like (Fn) type-3 domains. Notably, cMyBP-C contains an additional Ig domain (C0) at the extreme N-terminus, a phosphorylation motif in the M domain and an insertion within C5 (Moss et al., 2015; Sadayappan and de Tombe, 2012; Sequeira et al., 2014). The C-terminal domains are responsible for anchoring cMyBP-C to the thick filament, while the N-terminus is thought to be involved in the interaction with both myosin and actin (Moss et al., 2015; Sadayappan and de Tombe, 2012; Sequeira et al., 2014). Extensive cMyBP-C phosphorylation contributes to myosin head alignment and extension towards the thin filaments, thus producing strong actin–myosin interactions and enhanced contractile output (Moss et al., 2015; Sadayappan and de Tombe, 2012; Sequeira et al., 2014). Abnormalities in cMyBP-C are also associated with cardiac disease pathogenesis. Dephosphorylation of cMyBP-C enhances its dissociation from the sarcomere into the bulk cytoplasm (Govindan et al., 2012), a cMyBP-C feature that is observed in animal models of heart failure and pathologic hypertrophy (Sequeira et al., 2014). Moreover, dominant mutations in slow MyBP-C have been linked to the development of distal arthrogryposis (Geist and Kontrogianni-Konstantopoulos, 2016), while cMyBP-C mutations have been reported in hypertrophic cardiomyopathy (HCM), DCM and left ventricular non-compaction (Carrier et al., 2015).

Impaired muscle relaxation and the alteration of subcellular Ca^2+^ signalling are common features in several animal models with mutant cMyBP-C (Brickson et al., 2007; Pohlmann et al., 2007; Song et al., 2003), pointing to a role for cMyBP-C as a multifaceted signalling node in myocyte function that contributes to the assembly, stabilisation and regulation of sarcomere shortening, as well as to the critical modulation of Ca^2+^ homeostasis. Here, we present several lines of evidence that suggests cMyBP-C influences cardiomyocyte intracellular Ca^2+^ signalling via a novel stoichiometric interaction with RyR2 Ca^2+^ release channels.

## RESULTS

### Identification of cMyBP-C as a novel RyR2-binding protein

To identify novel RyR2-binding proteins, we employed the yeast two-hybrid (Y2H) system using an RyR2 N-terminal fragment (residues 1–906, BT4L; RyR2^NT^) as the ‘bait’ to screen a human heart cDNA library. Several positive clones were found and, through nucleotide sequence analysis, were shown to encode the C-terminus (residues 817–1274) of cMyBP-C (cMyBP-C^CT^). When the isolated cMyBP-C^CT^ clone was screened against a series of overlapping fragments covering the entire length of RyR2, a positive interaction was detected only for RyR2^NT^ (Table 1), demonstrating the RyR2 N-terminal (residues 1–906) domain-specificity of cMyBP-C^CT^ binding.

**Table 1.**
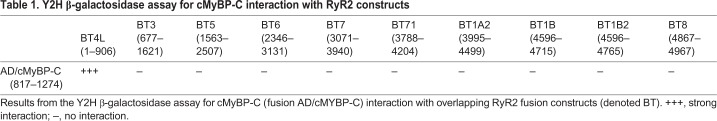
**Y2H β-galactosidase assay for cMyBP-C interaction with RyR2 constructs**

**Fig. 1.**
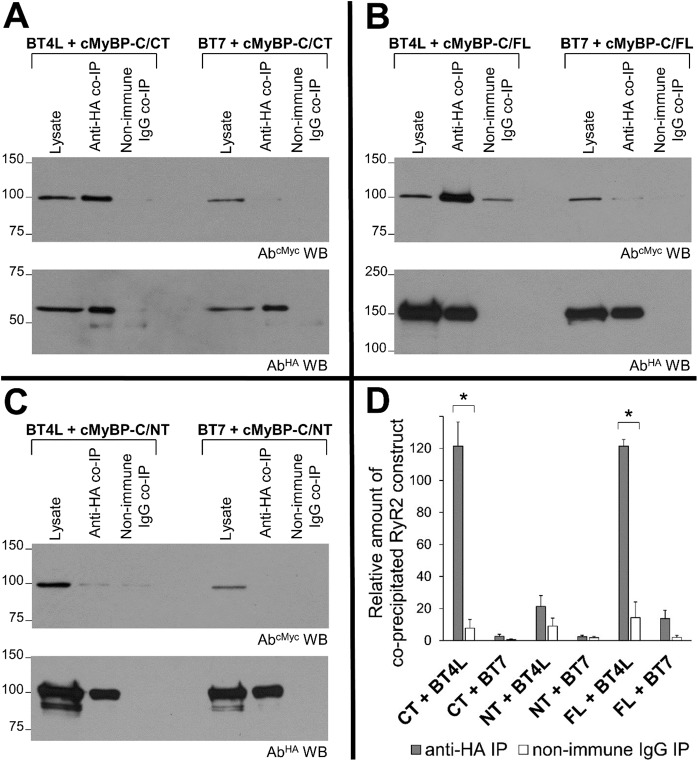
**Interaction between the cMyBP-C C-terminus and RyR2 N-terminus.** Co-IP assays from HEK293 cells co-expressing BT4L (cMyc-tagged RyR2 residues 1–906) or BT7 (cMyc-tagged RyR2 residues 3071–3940) together with HA-tagged cMyBP-C CT (residues 817–1274) (A), FL (full-length) (B), or NT (residues 1–866) (C). HA-cMyBP-C constructs were immunoprecipitated with Ab^HA^ from solubilised cell lysates and the presence of co-precipitated BT4L/BT7 was analysed by western blotting using Ab^cMyc^ (top). To detect immuno-isolated HA–cMyBP-C, 1/10th of the IP samples was analysed by western blotting using Ab^HA^ (bottom). Non-immune rabbit IgG served as negative control (non-specific binding). An aliquot of HEK293 cell lysate corresponding to 0.5% of the amount processed in the co-IP assay was included in the gels to assess protein expression. (D) Data summary (*n*≥5) following densitometry analysis and normalisation to the values in the lysate for each respective construct (taken as 100%); data are given as mean±s.e.m. *P<0.05 (paired two-tailed Student's *t*-test).

To reinforce our Y2H observations, putative binding of RyR2 to cMyBP-C was assessed by co-immunoprecipitation (co-IP) following co-expression of distinct fusion constructs in mammalian HEK293 cells, an immortalised cell lineage devoid of RyR2 and cMyBP-C. Thus, HA-tagged cMyBP-C^CT^ was immunoprecipitated using anti-HA antibody (Ab^HA^) and verified by immunoblotting (Fig. 1A, bottom panel), while the presence of co-precipitated cMyc-tagged RyR2^NT^ was analysed by immunoblotting with anti-Myc antibody (Ab^cMyc^). The co-IP revealed that RyR2^NT^ was recovered in the Ab^HA^ immunoprecipitate but not in the rabbit IgG control (Fig. 1A), suggesting that there is an interaction between the recombinant proteins RyR2^NT^ and cMyBP-C^CT^. Consistent with the Y2H data (Table 1), parallel co-IP experiments revealed no interaction of cMyBP-C with BT7 (Fig. 1A), a RyR2 central region construct (residues 3071–3940) of similar size to BT4L. RyR2^NT^ was also tested for an interaction either with full-length cMyBP-C (cMyBP-C^FL^) or a large N-terminal fragment comprising domains C0–C6 (NT, residues 1–865; cMyBP-C^NT^). Distinctive RyR2^NT^ co-precipitation was observed when co-expressed with cMyBP-C^FL^, but was negligible for cMyBP-C^NT^ (Fig. 1B,C). Densitometric analysis indicates equivalent specific binding of RyR2^NT^ with cMyBP-C^FL^ and cMyBP-C^CT^ (Fig. 1D). In contrast, RyR2^NT^ binding to cMyBP-C^NT^, as well as BT7 binding for any cMyBP-C construct, was minimal (Fig. 1D). These results indicate that RyR2 forms complexes with cMyBP-C *in vitro* via an interaction between the cMyBP-C C-terminus domain and the RyR2 N-terminus.

Given the high levels of sequence conservation across all three isoforms of MyBP-C C-termini (∼70%), as well as for the N-termini of all three RyR isoforms (>80%), the RyR1 and RyR3 N-terminal fragments [BT4LR1, amino acids 1–915 (RyR1^NT^) and BT4LR3, amino acids 1–911 (RyR3^NT^)] were also tested for cMyBP-C^CT^ binding in co-IP assays. Interaction of cMyBP-C^CT^ with both RyR1^NT^ and RyR3^NT^ was observed that was comparable to that with RyR2^NT^ (Fig. S2), suggesting that RyR–MyBP-C association may occur in both cardiac and skeletal muscle.

### Fibronectin-like type 3 domains mediate a robust RyR2–cMyBP-C interaction

**Fig. 2.**
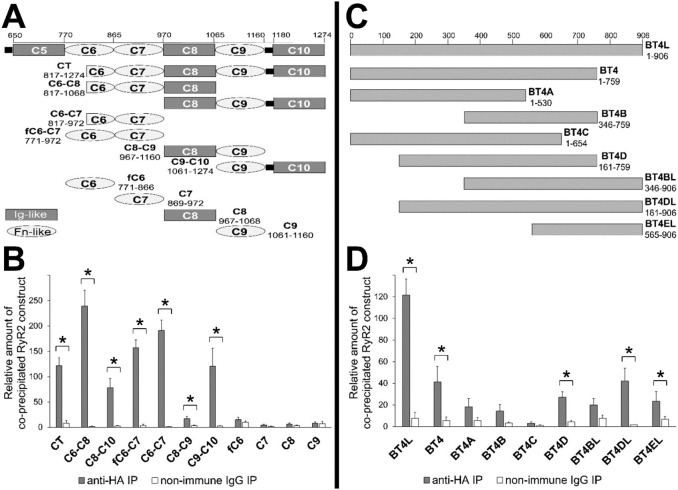
**Mapping of binding sites in RyR2 and cMyBP-C.** Schematic diagram of (HA-tagged) constructs covering the cMyBP-C C-terminus (A) and RyR2 N-terminus (Myc-tagged) subfragments (denoted BT) (C). Co-IP assays from HEK293 cells co-expressing BT4L with HA–cMyBP-C constructs (B), or cells co-expressing BT constructs with HA–cMyBP-C/CT (D). Data summary (*n*=numbers for each data set are: left, CT, 9; C6–C8, 3; C8–C10, 8; fC6–C7, 3; C6–C7, 4; C8–C9, 6; C9–C10, 4; fC6, 8; C7, 7; C8, 8; C9, 7; right, BT4L, 9; BT4, 4; BT4A, 7; BT4B, 7; 5, BT4C; 6, BT4D; BT4BL, 7, BT4DL, 4; BT4EL, 7) following quantitative densitometry analysis and normalisation to each construct's respective lysate (taken as 100%); data are given as mean±s.e.m. *P<0.05 (paired two-tailed Student's *t*-test).

To determine the RyR2-binding site within cMyBP-C^CT^, which comprises part of C6 and the C7–C10 domains (Fig. 2A), ten overlapping cMyBP-C^CT^ subfragments that had boundaries within linker sequences between discrete structural domains, to facilitate correct protein folding, were generated (Fig. 2A). Since the cMyBP-C^CT^ isolated in the Y2H screen contained only part of domain C6, cMyBP-C constructs encoding the whole C6 domain (fC6) were also prepared. Thus, co-IP assays (*n*≥5) with HEK293 cell lysates co-expressing HA-tagged cMyBP-C constructs and RyR2^NT^ (Fig. 2B) were undertaken. Both the overlapping triple-domain constructs, C6–C8 and C8–C10, displayed interaction with RyR2^NT^, with a ∼3-fold stronger signal for C6–C8 versus C8–C10. Further double-domain constructs C6–C7 and fC6–C7, C8–C9 and C9–C10 also exhibited RyR2^NT^ binding, providing the apparent rank order; C6–C8≈C6–C7>C8–C10≈C9–C10>>C8–C9. These results suggest that there is an Fn domain requirement for the cMyBP–RyR2^NT^ interaction, in particular for C7 and then for C9, which share 36% sequence identity. Domain C6, which appears to be dispensable, since cMyBP-C^NT^ exhibited minimal RyR2^NT^ binding, has lower sequence identity with C7 and C9 (22% and 24%, respectively). Notably, individual domains C7 and C9 (as well as fC6, C8) displayed negligible RyR2^NT^ binding, suggesting that two domains are minimally required either to comprise the appropriate conformation or to jointly contribute to the RyR2-binding interface.

Similarly, to further map the cMyBP-C binding site within the RyR2 N-terminus, a series of eight overlapping cMyc-tagged RyR2^NT^ subfragments (Fig. 2C) were co-expressed together with cMyBP-C^CT^ in HEK293 cells for co-IP assays. Quantitative (*n*≥5) densitometry and statistical analysis indicates that RyR2^NT^ subfragments BT4 (residues 1–759), BT4D (residues 161–759), BT4DL (residues 161–906) and BT4EL (residues 565–906) retain significant cMyBP-C binding (compared to their respective non-specific binding, *P*<0.05), although binding was reduced relative to RyR2^NT^ (Fig. 2D). These results suggest multiple determinants within the RyR2 N-terminus are necessary for robust interaction with cMyBP-C.

### Native porcine cardiac and recombinant human RyR2–cMyBP-C association

**Fig. 3.**
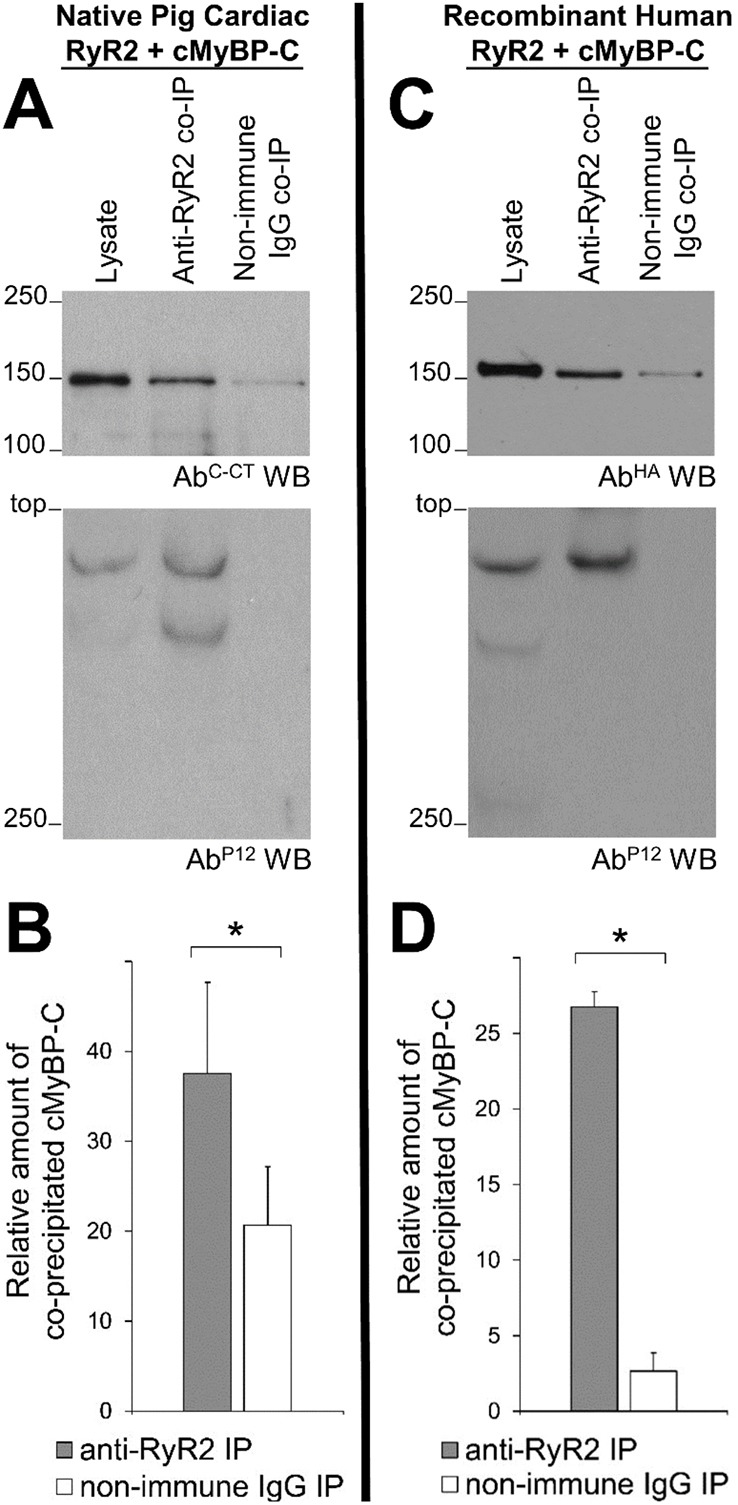
**Native pig and recombinant human RyR2–cMyBP-C interaction.** Co-IP assays from pig cardiac tissue (A) or HEK293 cells co-expressing full-length human RyR2 with human cMyBP-C/FL (C). RyR2 was immunoprecipitated with Ab^1093^ from solubilised pig cardiac (A) or HEK293 cell lysate (C), and the presence of co-precipitated cMyBP-C was analysed by western blotting using Ab^C-CT^ or Ab^HA^, respectively (top). To detect immuno-isolated RyR2, 1/10th of the IP samples was analysed by western blotting using Ab^P12^ (bottom). Non-immune rabbit IgG served as negative control (non-specific binding). An aliquot of cell lysate corresponding to 0.5% of the amount processed in the co-IP assay was included in the gels to assess protein expression. (B,D) Data summary (*n*=5) following densitometry analysis and normalisation to the lysate (taken as 100%); data are given as mean value±s.e.m. *P<0.05 (paired two-tailed Student's *t*-test).

To further confirm the cMyBP-C^CT^ interaction with RyR2^NT^ that was seen in the heterologous expression system (yeast), we sought to identify the putative RyR2–cMyBP-C association in native mammalian tissue. Initially, we investigated cMyBP-C expression in the SR compartment, using porcine cardiac SR and antibodies raised against either the cMyBP-C N-terminus or C-terminus (Ab^C-NT^ and Ab^C-CT^, respectively; Fig. S3). Following subcellular fractionation, a proportion of the soluble cMyBP-C was detected in the cytosol. Importantly, substantial amounts of cMyBP-C co-migrated with the SR fraction suggesting association with an SR-resident moiet(ies), potentially RyR2. To address such a possibility, we performed RyR2 co-IP experiments using solubilised pig cardiac tissue (with RyR2-specific Ab^1093^) and co-precipitated cMyBP-C was monitored (using Ab^C-CT^; Fig. 3A,B). We determined that cMyBP-C was readily detected upon RyR2 immuno-isolation, with statistically significant specific binding between the native proteins (*P*<0.05).

**Fig. 4.**
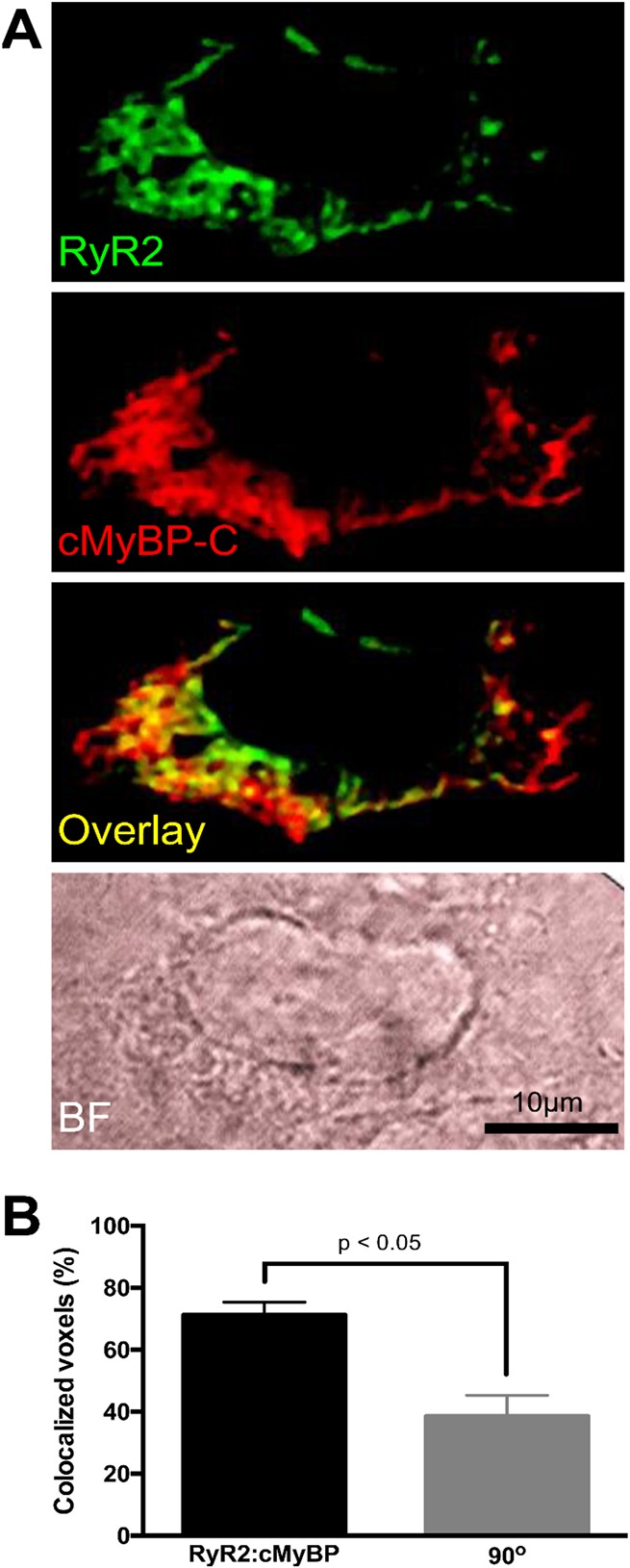
**Subcellular colocalisation of RyR2 with cMyBP-C in HEK293 cells.** (A) Representative images of the intracellular distribution of heterologous RyR2 (green) and cMyBP-C (red) in a single HEK293 cell. Composite images were generated in which spatially colocalised voxels corresponding to RyR2 and cMyBP-C were overlaid (yellow). The corresponding bright-field image of the cell is also given (BF). (B) The extent of RyR2 colocalisation with cMyBP-C was quantified. Non-specific colocalisation was assessed following geometric rotation of the cMyBP-C image through 90° (also see Fig. S4). The data on RyR2–cMyBP-C colocalisation are plotted as mean±s.e.m. and are from analysis of 192 cells from four separate experiments. The values resulting from the rotation of cMyBP-C images through 90° are given as mean±s.e.m. and are from the analysis of 10 randomly selected images. *P<0.05 (paired two-tailed Student's *t*-test).

Parallel experiments using HEK293 cells co-expressing both the human proteins further demonstrated significant (*P*<0.05) specific interaction between the recombinant human RyR2 and cMyBP-C (Fig. 3C,D). Moreover, the co-immunolocalisation observed for recombinant human RyR2 and cMyBP-C is consistent with potential subcellular interaction between these proteins (Fig. 4A). The proportion of RyR2-positive voxels that directly colocalised with cMyBP-C was 71.2±4.1%, a significantly greater value than for non-specific colocalisation obtained via geometric rotation of the cMyBP-C image through 90° (38.6±6.7%, mean±s.e.m., *P*<0.05) (Fig. 4B; Fig. S4). The extent of colocalisation between RyR2 and cMyBP-C was independent of the immunofluorescence signal intensity corresponding to RyR2 expression levels (Fig. S4).

### cMyBP-C modulates RyR2-mediated cellular Ca^2+^ dynamics

**Fig. 5.**
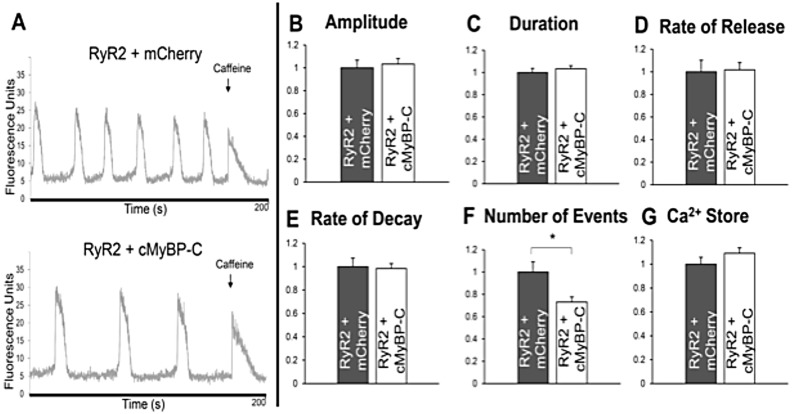
**cMyBP-C alters RyR2-mediated Ca^2+^ handling in HEK293 cells.** Single-cell Ca^2+^ imaging performed via confocal laser scanning microscopy to monitor spontaneous intracellular Ca^2+^ mobilisation. Representative traces (left, A) and summary of results (B–G) from cells expressing RyR2 plus mC–cMyBP-C (*n*=70), or RyR2 plus mCherry (*n*=33). To estimate cell ER load, 10 mM caffeine was added after 2 min 30 s and data were recorded for a further 30 s. Parameters of Ca^2+^ release events analysed include amplitude (B), duration (C), rate of release (D), rate of decay (E), frequency (F) and Ca^2+^ store content (G). Data are normalised for RyR2 alone and are presented as mean±s.e.m. *P<0.05 [one-way ANOVA with Bonferroni's multiple comparisons test (B–E) or Kruskal–Wallis test with Dunn's multiple comparisons test (F,G)].

To ascertain whether cMyBP-C expression influences RyR2-mediated intracellular Ca^2+^ handling, spontaneous Ca^2+^ release events in HEK293 cells were monitored through confocal imaging. N-terminal tagging of cMyBP-C with mCherry (mC–cMyBP-C), which is spectrally distinct from fluo-3 Ca^2+^-reporting dye, enabled visualisation of cells expressing mC–cMyBP-C. Parallel experiments were completed with two separate cell populations expressing: (1) RyR2 and mC-cMyBP-C, and (2) RyR2 and mCherry (Fig. 5A). The Ca^2+^ transient amplitude (Fig. 5B), Ca^2+^ transient duration (Fig. 5C), rate of Ca^2+^ release (Fig. 5D), Ca^2+^ decay rate (Fig. 5E) and endoplasmic reticulum (ER) Ca^2+^ store content (Fig. 5G) were unaffected by the presence of mC–cMyBP-C or mCherry. The specific distinction between cells expressing RyR2 plus mC-cMyBP-C, relative to RyR2 plus mCherry, was a substantial and statistically significant decrease in the number of spontaneous Ca^2+^ oscillations (*P*<0.05, Fig. 5F). This reduced propensity for spontaneous RyR2-mediated Ca^2+^ release in the specific presence of mC–cMyBP-C would be consistent with an inhibitory role for cMyBP-C on RyR2 channel function.

## DISCUSSION

**Fig. 6.**
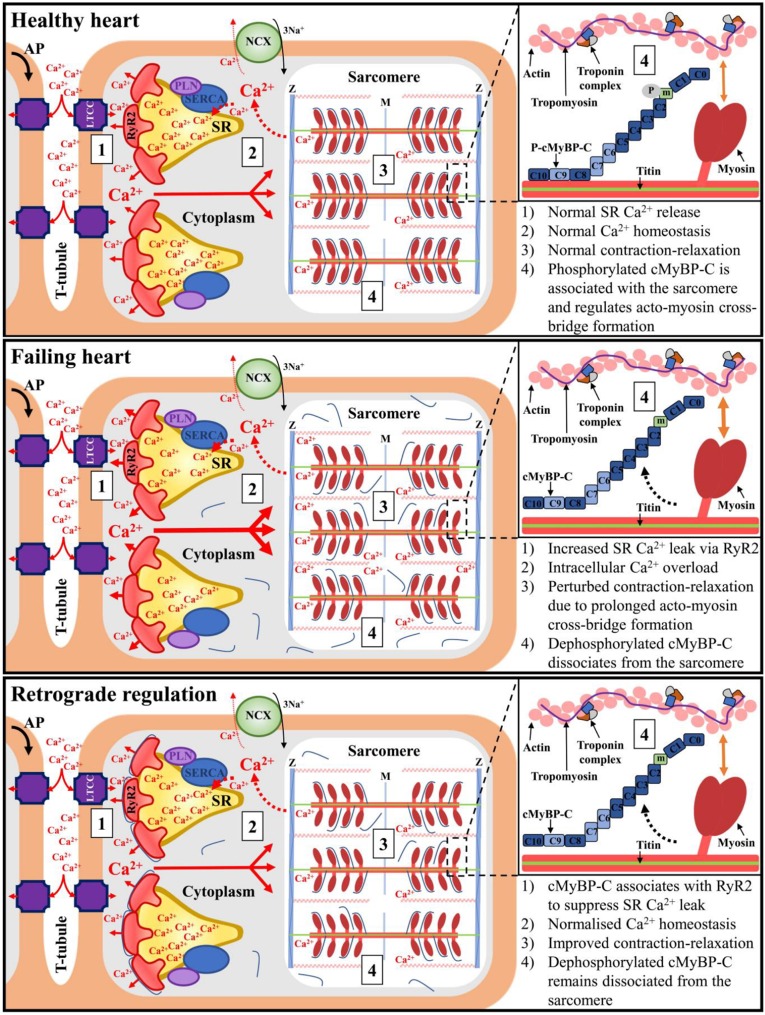
**Working hypothesis for retrograde regulation of SR Ca^2+^ release by the sarcomere.** In the healthy heart, an action potential triggers a transient rise in the intracellular Ca^2+^ concentration, which in turn induces sarcomere contraction. cMyBP-C is in its phosphorylated form and predominantly associated with myofilaments, where it regulates acto-myosin cross-bridge formation. In pathological conditions associated with SR Ca^2+^ leak (Failing heart) leading to intracellular Ca^2+^ overload, dephosphorylated cMyBP-C translocates from the sarcomere to the SR, where it suppresses Ca^2+^ leak through its interaction with RyR2 (Retrograde regulation) facilitating normal Ca^2+^ homeostasis and contraction. AP, action potential; LTCC, L-type Ca^2+^ channel; NCX: Na^+^/Ca^2+^ exchanger; PLN, phospholamban; SERCA, SR/ER Ca^2+^ ATPase.

Excitation–contraction coupling is considered to be a unidirectional process where the rise in intracellular Ca^2+^ concentration triggered by an action potential induces sarcomere contraction. Given that dephosphorylated cMyBP-C is released from the sarcomere in pathological conditions (Baker et al., 2015; Decker et al., 2012; Govindan et al., 2012; Kulikovskaya et al., 2007; Kuster et al., 2014), the structural and functional interaction between the SR-resident RyR2 and sarcomere-released, soluble cMyBP-C, described in the present study, may therefore constitute an important, and previously unrecognised, feedback regulatory mechanism (Fig. 6). Our initial observation of an interaction between the RyR2 N-terminus and the cMyBP-C C-terminus provided from an unbiased transcriptome-wide screen, was further verified by biochemical assays (Fig. 1). Parallel experiments with the corresponding N-terminal fragment from skeletal muscle isoform RyR1 indicated a robust interaction with cMyBP-C (Fig. S2), suggesting that MyBP-C binding might be relevant not only in cardiac, but also in skeletal muscle and smooth muscle. Importantly, porcine cMyBP-C was found to associate with the cardiac SR subcellular endomembranous compartment (Fig. S3) and to co-immunoprecipitate with native porcine RyR2 (Fig. 3). Moreover, human cMyBP-C colocalised with RyR2 in HEK293 cells (Fig. 4), co-immunoprecipitated with human RyR2 (Fig. 3) and modulated spontaneous RyR2 Ca^2+^ release (Fig. 5). That cMyBP-C dephosphorylation is required for sarcomere dissociation (Govindan et al., 2012) suggests that dephosphorylated cMyBP-C is the form that interacts with RyR2. As the functional phosphorylation sites of cMyBP-C are at the N-terminus (Moss et al., 2015), while the RyR2 interaction site occurs via the C-terminus, it appears unlikely that cMyBP-C phosphorylation would have a direct impact on RyR2 association. However, the precise role of cMyBP-C phosphorylation status on specific binding with RyR2 and its pathophysiological significance remains to be resolved.

To our knowledge, these observations constitute the first report that suggests a direct role may exist for cMyBP-C in the regulation of RyR2-mediated Ca^2+^ homeostasis. Our data are consistent with previous indirect evidence from transgenic mouse models showing that an altered cMyBP-C protein is linked to the prolongation of Ca^2+^ release events (Brickson et al., 2007; Pohlmann et al., 2007; Song et al., 2003). While this effect of an altered cMyBP-C could be explained by increased myofilament Ca^2+^ sensitivity (Huke and Knollmann, 2010), a plausible alternative or additional mechanism may involve a role for cMyBP-C modulation of a crucial Ca^2+^ signalling component.

The rate of Ca^2+^ transient decay is governed by the multilateral involvement of: (1) the SR/ER Ca^2+^/ATPase (SERCA) pumping Ca^2+^ into the SR, (2) phospholamban inhibiting SERCA activity, which could be relieved following phospholamban phosphorylation, (3) the Na^+^/Ca^2+^ exchanger (NCX) extruding Ca^2+^ out of the cell, and (4), the SR Ca^2+^ leak through the RyR2, which could be substantial in pathological conditions (Bers, 2002; Bers et al., 2003; George, 2008). The relative contribution of SERCA is ∼92%, whereas that of NCX is ∼7% for diastolic Ca^2+^ removal in the mouse heart (Bers, 2002). In cMyBP-C knockout mice, RyR2 expression and/or channel activity was not investigated but the level of NCX activity was 34% lower (Pohlmann et al., 2007), which could partly account for the prolonged Ca^2+^ transient observed. However, the level of phosphorylated phospholamban was 3-fold higher in knockout mouse hearts (with unchanged SERCA and phospholamban protein levels) (Pohlmann et al., 2007), and therefore a reduction in Ca^2+^ transient duration would be predicted rather than an increase. Given the 13:1 SERCA (∼92%):NCX (∼7%) contribution to diastolic Ca^2+^ removal, the prolonged Ca^2+^ transient seen in cMyBP-C knockout mice appears highly unlikely to be solely due to 34% lower NCX levels – a notable alternative/additional contribution may be the involvement of SR Ca^2+^ leak through the RyR2. In this scenario, it could be postulated that cMyBP-C is normally required to suppress diastolic SR Ca^2+^ leak through its interaction with RyR2 (Fig. 6). The putative suppression of RyR2 channel activity by cMyBP-C, interpreted from electrically stimulated Ca^2+^ transients in cardiomyocytes, is entirely consistent with our cMyBP-C co-expression results showing a reduced propensity for RyR2-mediated spontaneous Ca^2+^ transients in a mammalian cell line (Fig. 5), although Ca^2+^ release event duration was unaltered.

Formation of the RyR2–cMyBP-C complex in cardiomyocytes will depend on the cMyBP-C subcellular distribution. As part of the physiological sarcomeric protein turnover, cMyBP-C is known to exist as two different forms *in vivo*, the predominant phosphorylated form (associated with greater thick filament stability) and a second dephosphorylated form (Moss et al., 2015; Sadayappan and de Tombe, 2012). The latter is relatively sensitive to release from the sarcomere, with low levels of full-length cMyBP-C reported in the cytosol (Baker et al., 2015; Decker et al., 2012; Kuster et al., 2014), which is in agreement with our own observations (Fig. S3). Notably, an increased rate of cMyBP-C dephosphorylation, and its subsequent dissociation from the sarcomere and further degradation, has been reported in the heart of animal models and patients with heart failure, myocardial infarction, ischaemia–reperfusion injury and pathological hypertrophy (Baker et al., 2015; Decker et al., 2012; Govindan et al., 2012; Kulikovskaya et al., 2007; Kuster et al., 2014). Interestingly, high-resolution imaging of cMyBP-C and RyR2 revealed that sarcomere-bound cMyBP-C is the predominant form in healthy cardiomyocytes (Previs et al., 2015), with significant release of cMyBP-C into the cytosol after myocardial infarction/reperfusion (Govindan et al., 2012) and in pathologic hypertrophy (Sequeira et al., 2014). Thus, it is plausible that sarcomere-dissociated cMyBP-C may be available for RyR2 interaction and modulation of SR Ca^2+^ release, particularly in the above cardiopathologic conditions (Fig. 6).

The RyR2:cMyBP-C association could also be relevant in DCM and HCM, cardiomyopathies often accompanied by an arrhythmogenic phenotype (Maron et al., 2014; Spezzacatene et al., 2015). RyR2 mutations including those within the N-terminus have recently been linked to DCM (Haas et al., 2014), whereas mutations in cMyBP-C are very common in both HCM and DCM pathology (Carrier et al., 2015), with some cMyBP-C mutations reported to trigger arrhythmic events (Bahrudin et al., 2008; Berul et al., 2001). A proportion of mutants encode for cMyBP-C truncated protein(s) that lack the C-terminal C10 domain, a structural element critical for association with the thick filament (Flashman et al., 2007) but a domain entirely dispensable for RyR2 binding (Fig. 2). It should be noted though that the likely pathogenic mechanism of such mutations is haploinsufficiency, since the predicted cMyBP-C truncated polypeptides could not be detected in human patient samples (Carrier et al., 2015; Sequeira et al., 2014). Proteins with missense mutations (e.g. A31P and E258K) are likely to be expressed and incorporated into the myofilaments (De Lange et al., 2013; Harris et al., 2011), whereas a 25-nucleotide frameshift deletion resulting in a novel sequence within the C10 domain exhibits little incorporation into the C-zone of the sarcomere and localises at the Z-line (Kuster et al., 2015) – a specific location where the RyR2 is found. Interestingly, cMyBP-C mutations resulting in truncation have been mostly associated with HCM, whereas missense mutations are mostly linked with DCM (Waldmuller et al., 2011). Two different mechanisms underlying increased myofilament Ca^2+^ sensitivity and contractile dysfunction have previously been proposed for cMyBP-C truncations and missense mutations, respectively: (1) haploinsufficiency that might also involve impairment of nonsense-mediated mRNA decay and/or the ubiquitin-proteasome system, and (2) dominant-negative mutant proteins acting as poison polypeptides (Carrier et al., 2015; Harris et al., 2011; Sequeira et al., 2014). Our study raises the possibility that perturbation of Ca^2+^ homeostasis due to altered RyR2–cMyBP-C interactions may constitute an additional pathogenic mechanism contributing to the development of DCM, HCM and/or arrhythmogenicity. Future studies are required to unravel the precise molecular mechanism(s) by which MyBP-Cs are involved in, possibly dynamic, RyR isoform-specific regulation of SR Ca^2+^ release and to fully elucidate the role of RyR2–cMyBP-C association in cardiac physiology and disease.

## MATERIALS AND METHODS

### Materials

Mammalian cell culture reagents and Fluo-3 AM were obtained from Thermo Scientific, herring testes carrier DNA from Takara, protease inhibitor cocktail (Complete™) from Roche, Protein-A–Sepharose from GE Healthcare, electrophoresis equipment and reagents from Bio-Rad, enhanced chemiluminescence detection kit from Thermo Scientific, DNA restriction endonucleases from New England Biolabs and the Pfu DNA polymerase from Promega; oligonucleotides and all other reagents were from Sigma unless otherwise stated.

### Antibodies

Primary antibodies used were: rabbit RyR2-specific Ab^1093^ raised against residues 4454–4474 of human RyR2 [used in co-immunoprecipitation (co-IP) at 2 µg and immunofluorescence (IF) at 1:100 dilution] as previously described (Zissimopoulos et al., 2012, 2006), mouse RyR2 Ab^P12^ raised against residues 2846–2859 of human RyR2 [used in western blotting (WB) at 1:500 dilution, see Fig. S1], sheep cMyBP-C-specific Ab^C-CT^ (AF7199, R&D Systems) raised against mouse cMyBP-C residues 998–1100 (used in WB at 1:200 dilution), mouse cMyBP-C-specific Ab^C-NT^ (E7, Santa Cruz Biotechnology) raised against residues 1–120 of human cMyBP-C (used in WB at 1:500 dilution and IF at 1:100 dilution), rabbit Ab^HA^ (Y-11, Santa Cruz Biotechnology; used in co-IP at 2 μg per reaction), non-immune rabbit IgG (Santa Cruz Biotechnology; used in co-IP at 2 μg), mouse Ab^HA^ [16B12, Covance (BioLegend); used in WB at 1:1000 dilution], mouse Ab^cMyc^ (9E10, Santa Cruz Biotechnology; used in WB at 1:500 dilution). Secondary antibodies used were: goat anti-mouse IgG-horseradish peroxidase conjugate (Santa Cruz Biotechnology; used in WB at 1:10,000 dilution), donkey anti-sheep IgG-horseradish peroxidase conjugate (R&D Systems; used in WB at 1:5000 dilution), goat anti-rabbit IgG-Alexa Fluor 488 conjugate (A-11034, Thermo Scientific; used in IF at 1:500 dilution) and goat anti-mouse IgG-Alexa Fluor 594 conjugate (A-11005, Thermo Scientific; used in IF at 1:500 dilution).

### Plasmid constructs

Plasmids encoding for RyR2 overlapping fragments (BT constructs) tagged with the cMyc epitope at the N-terminus were as previously described (Zissimopoulos et al., 2013). mCherry cDNA was obtained from Addgene (plasmid #30125) and cloned into the mammalian expression vector pCR3 (Thermo Scientific). Human cMyBP-C cDNA was generated by PCR amplification from a human heart cDNA library (Takara) and cloned into pCR3 containing an N-terminal HA epitope tag or mCherry fusion protein. Smaller HA-tagged cMyBP-C constructs were generated by PCR amplification and cloned in the HA-modified pCR3 vector. Oligonucleotide primers and restriction enzyme cloning sites used are given in Table S1.

### Mammalian cell culture

HEK293 cells (ATCC) were cultured in Dulbecco's modified Eagle's medium supplemented with 2 mM glutamine and 10% fetal bovine serum (FBS) in a 37°C humidifying incubator with 5% CO_2_. For use in co-IP, ∼1.5×10^6^ cells were seeded in one 100 mm Petri dish a day before transfection in order to be 60–70% confluent the following day. Cells were transfected with 24 μg of plasmid DNA using the calcium phosphate precipitation method as described elsewhere (Stanczyk et al., 2016). For use in IF, ∼0.25×10^6^ cells were seeded onto poly-lysine-coated glass coverslips (22 mm×22 mm), left to adhere overnight and transfected the following day using TurboFect (Thermo Scientific) according to the manufacturer's instructions. For Ca^2+^ imaging, ∼1×10^5^ cells were seeded on poly-lysine coated glass bottom dishes (MatTek) and transfected using Effectene (Qiagen) according to the manufacturer's instructions.

### Immunofluorescent detection of RyR2 and cMyBP-C

Transfected HEK293 cells were washed in phosphate-buffered saline (PBS) (137 mM NaCl, 2.7 mM KCl, 10 mM Na_2_HPO_4_, 1.8 mM KH_2_PO_4_ pH 7.4) and fixed with 4% paraformaldehyde for 10 min at room temperature. Fixed cells were washed and re-hydrated with PBS for 1 h before permeabilisation with 0.1% Triton X-100 for 30 min. Cells were washed with PBS, blocked with 10% FBS for 1 h, and then incubated at room temperature for 2 h with rabbit RyR2-specific Ab^1093^ and mouse cMyBP-C-specific Ab^C-NT^ antibodies. Samples were then washed with PBS and incubated with Alexa Fluor 488- and Alexa Fluor 594-conjugated goat anti-rabbit-IgG and goat anti-mouse-IgG antibodies for fluorescent labelling of RyR2 and cMyBP-C, respectively, at room temperature for 2 h in the dark. Following final washing with PBS, the coverslips were washed in water, dried and mounted onto glass slides using ProLong Gold Antifade reagent (Thermo Scientific) and stored at 4°C until required. All antibodies were carefully titrated to give comparable fluorescence signals under test. There was negligible non-specific fluorescence in these cells as determined by using the staining protocol as above but with one or both primary and secondary antibodies omitted.

### Image analysis and processing

Immunofluorescence signals corresponding to RyR2 and cMyBP-C were visualised using a confocal microscope (SP5, Leica Microsystems) fitted with a 63× oil immersion objective (NA 1.4). Signal intensity in the images were adjusted to the full dynamic range using the operating software's ‘glow-over’ function (LAS-AF, Leica Microsystems) and were collected throughout the depth of each samples (*z*-stack 0.5 µm slices, 18.5±0.24 frames, and average depth of cell 8.6±1.2 µm). Individual *z*-plane images were recombined into three-dimensional datasets using LAS-AF (Leica Microsystems). Data were imported into Imaris (Bitplane, Andor Technology) and the signal threshold for bona fide RyR2 and cMyBP-C-positive voxels in each image was defined using the levels of non-specific immunofluorescence established as above. Over the entire dataset (*n*=192 3D reconstructions from four separate transfections), RyR2- and cMyBP-C-positive voxels were assigned to 2.8±0.1% and 7.8±0.3% (mean±s.e.m.) of the total voxels, respectively. In each *z*-stack, the proportion of RyR2 voxels that were spatially co-incident with cMyBP-C voxels was calculated using Imaris software. Normally distributed data were subjected to statistical testing using unpaired two-tailed Student's *t*-test and non-normally distributed data were tested using an unpaired Mann–Witney test. Linear regression and the calculation of Pearson's correlation coefficient (PCC) was performed (Microsoft Excel, Office 2016). *P*<0.05 was taken as significant.

### Other methodologies

The Y2H system (Lam et al., 2013; Stanczyk et al., 2016; Zissimopoulos et al., 2006), co-IP and WB (Stanczyk et al., 2016; Zissimopoulos et al., 2012, 2013), cardiac SR preparation (Zissimopoulos et al., 2012) and single-cell Ca^2+^ imaging (Handhle et al., 2016; Seidel et al., 2015b) were carried out as previously described. Densitometry analysis was carried out using GS700 densitometer (Bio-Rad) and Quantity-one (Bio-Rad) software. Microsoft Excel was used to store and plot numerical data (expressed as mean±s.e.m.), unless otherwise stated. Statistical analysis was performed using GraphPad prism (GraphPad Software Inc). Data were first subjected to the Brown–Forsythe test for the equality of group variances, and populations with equal variance (no significantly different standard deviations, *P*<0.05) were analysed using one-way ANOVA (with Bonferroni's post-test). Where data were non-normally distributed, the Kruskal–Wallis test (with Dunn's multiple comparisons test) was used.

## Supplementary Material

Supplementary information

## References

[JCS210443C1] Bahrudin U., Morisaki H., Morisaki T., Ninomiya H., Higaki K., Nanba E., Igawa O., Takashima S., Mizuta E., Miake J. et al. (2008). Ubiquitin-proteasome system impairment caused by a missense cardiac myosin-binding protein C mutation and associated with cardiac dysfunction in hypertrophic cardiomyopathy. *J. Mol. Biol.* 384, 896-907. 10.1016/j.jmb.2008.09.070 18929575

[JCS210443C2] Baker J. O., Tyther R., Liebetrau C., Clark J., Howarth R., Patterson T., Möllmann H., Nef H., Sicard P., Kailey B. et al. (2015). Cardiac myosin-binding protein C: a potential early biomarker of myocardial injury. *Basic Res. Cardiol.* 110, 23 10.1007/s00395-015-0478-5 25837837 PMC4383815

[JCS210443C3] Bers D. (2002). Cardiac excitation-contraction coupling. *Nature* 415, 198-205. 10.1038/415198a 11805843

[JCS210443C4] Bers D. M., Eisner D. A. and Valdivia H. H. (2003). Sarcoplasmic reticulum Ca^2+^ and heart failure: Roles of diastolic leak and Ca^2+^ transport. *Circ. Res.* 93, 487-490. 10.1161/01.RES.0000091871.54907.6B 14500331

[JCS210443C5] Berul C. I., McConnell B. K., Wakimoto H., Moskowitz I. P. G., Maguire C. T., Semsarian C., Vargas M. M., Gehrmann J., Seidman C. E. and Seidman J. G. (2001). Ventricular arrhythmia vulnerability in cardiomyopathic mice with homozygous mutant Myosin-binding protein C gene. *Circulation* 104, 2734-2739. 10.1161/hc4701.099582 11723028

[JCS210443C6] Brickson S., Fitzsimons D. P., Pereira L., Hacker T., Valdivia H. and Moss R. L. (2007). In vivo left ventricular functional capacity is compromised in cMyBP-C null mice. *Am. J. Physiol. Heart Circ. Physiol.* 292, H1747-H1754. 10.1152/ajpheart.01037.2006 17122190

[JCS210443C7] Carrier L., Mearini G., Stathopoulou K. and Cuello F. (2015). Cardiac myosin-binding protein C (MYBPC3) in cardiac pathophysiology. *Gene* 573, 188-197. 10.1016/j.gene.2015.09.008 26358504 PMC6660134

[JCS210443C8] Clark K. A., McElhinny A. S., Beckerle M. C. and Gregorio C. C. (2002). Striated muscle cytoarchitecture: an intricate web of form and function. *Annu. Rev. Cell Dev. Biol.* 18, 637-706. 10.1146/annurev.cellbio.18.012502.105840 12142273

[JCS210443C9] Decker R. S., Nakamura S., Decker M. L., Sausamuta M., Sinno S., Harris K., Klocke F. J., Kulikovskaya I. and Winegrad S. (2012). The dynamic role of cardiac myosin binding protein-C during ischemia. *J. Mol. Cell. Cardiol.* 52, 1145-1154. 10.1016/j.yjmcc.2012.01.006 22281395

[JCS210443C10] De Lange W. J., Grimes A. C., Hegge L. F., Spring A. M., Brost T. M. and Ralphe J. C. (2013). E258K HCM-causing mutation in cardiac MyBP-C reduces contractile force and accelerates twitch kinetics by disrupting the cMyBP-C and myosin S2 interaction. *J. Gen. Physiol.* 142, 241-255. 10.1085/jgp.201311018 23980194 PMC3753599

[JCS210443C11] Flashman E., Watkins H. and Redwood C. (2007). Localization of the binding site of the C-terminal domain of cardiac myosin-binding protein-C on the myosin rod. *Biochem. J.* 401, 97-102. 10.1042/BJ20060500 16918501 PMC1698665

[JCS210443C12] Geist J. and Kontrogianni-Konstantopoulos A. (2016). MYBPC1, an emerging myopathic gene: what we know and what we need to learn. *Front. Physiol.* 7, 410 10.3389/fphys.2016.00410 27683561 PMC5021714

[JCS210443C13] George C. H. (2008). Sarcoplasmic reticulum Ca2+ leak in heart failure: mere observation or functional relevance? *Cardiovasc. Res.* 77, 302-314. 10.1093/cvr/cvm006 18006486

[JCS210443C14] Govindan S., McElligott A., Muthusamy S., Nair N., Barefield D., Martin J. L., Gongora E., Greis K. D., Luther P. K., Winegrad S. et al. (2012). Cardiac myosin binding protein-C is a potential diagnostic biomarker for myocardial infarction. *J. Mol. Cell. Cardiol.* 52, 154-164. 10.1016/j.yjmcc.2011.09.011 21971072 PMC3246118

[JCS210443C15] Haas J., Frese K. S., Peil B., Kloos W., Keller A., Nietsch R., Feng Z., Muller S., Kayvanpour E., Vogel B. et al. (2014). Atlas of the clinical genetics of human dilated cardiomyopathy. *Eur. Heart J.* 36, 1123-1135. 10.1093/eurheartj/ehu301 25163546

[JCS210443C16] Handhle A., Ormonde C. E., Thomas N. L., Bralesford C., Williams A. J., Lai F. A. and Zissimopoulos S. (2016). Calsequestrin interacts directly with the cardiac ryanodine receptor luminal domain. *J. Cell Sci.* 129, 3983-3988. 10.1242/jcs.191643 27609834 PMC5117208

[JCS210443C17] Harris S. P., Lyons R. G. and Bezold K. L. (2011). In the thick of it: HCM-causing mutations in myosin binding proteins of the thick filament. *Circ. Res.* 108, 751-764. 10.1161/CIRCRESAHA.110.231670 21415409 PMC3076008

[JCS210443C18] Huke S. and Knollmann B. C. (2010). Increased myofilament Ca2+-sensitivity and arrhythmia susceptibility. *J. Mol. Cell. Cardiol.* 48, 824-833. 10.1016/j.yjmcc.2010.01.011 20097204 PMC2854218

[JCS210443C19] Kulikovskaya I., McClellan G. B., Levine R. and Winegrad S. (2007). Multiple forms of cardiac myosin-binding protein C exist and can regulate thick filament stability. *J. Gen. Physiol.* 129, 419-428. 10.1085/jgp.200609714 17470661 PMC2154376

[JCS210443C20] Kuster D. W. D., Cardenas-Ospina A., Miller L., Liebetrau C., Troidl C., Nef H. M., Mollmann H., Hamm C. W., Pieper K. S., Mahaffey K. W. et al. (2014). Release kinetics of circulating cardiac myosin binding protein-C following cardiac injury. *Am. J. Physiol. Heart Circ. Physiol.* 306, H547-H556. 10.1152/ajpheart.00846.2013 24337456 PMC3920245

[JCS210443C21] Kuster D. W. D., Govindan S., Springer T. I., Martin J. L., Finley N. L. and Sadayappan S. (2015). A hypertrophic cardiomyopathy-associated MYBPC3 mutation common in populations of South Asian descent causes contractile dysfunction. *J. Biol. Chem.* 290, 5855-5867. 10.1074/jbc.M114.607911 25583989 PMC4342493

[JCS210443C22] Lam A. K., Galione A., Lai F. A. and Zissimopoulos S. (2013). Hax-1 identified as a two-pore channel (TPC)-binding protein. *FEBS Lett.* 587, 3782-3786. 10.1016/j.febslet.2013.10.031 24188827

[JCS210443C23] Lanner J. T., Georgiou D. K., Joshi A. D. and Hamilton S. L. (2010). Ryanodine receptors: structure, expression, molecular details, and function in calcium release. *Cold Spring Harb. Perspect. Biol.* 2, a003996 10.1101/cshperspect.a003996 20961976 PMC2964179

[JCS210443C24] Maron B. J., Ommen S. R., Semsarian C., Spirito P., Olivotto I. and Maron M. S. (2014). Hypertrophic cardiomyopathy: present and future, with translation into contemporary cardiovascular medicine. *J. Am. Coll. Cardiol.* 64, 83-99. 10.1016/j.jacc.2014.05.003 24998133

[JCS210443C25] Moss R. L., Fitzsimons D. P. and Ralphe J. C. (2015). Cardiac MyBP-C regulates the rate and force of contraction in mammalian myocardium. *Circ. Res.* 116, 183-192. 10.1161/CIRCRESAHA.116.300561 25552695 PMC4283578

[JCS210443C26] Pohlmann L., Kroger I., Vignier N., Schlossarek S., Kramer E., Coirault C., Sultan K. R., El-Armouche A., Winegrad S., Eschenhagen T. et al. (2007). Cardiac myosin-binding protein C is required for complete relaxation in intact myocytes. *Circ. Res.* 101, 928-938. 10.1161/CIRCRESAHA.107.158774 17823372

[JCS210443C38] Previs M. J., Prosser B. L., Mun J. Y., Previs S. B., Gulick J., Lee K., Robbins J., Craig R., Lederer W. J. and Warshaw D. M. (2015). Myosin-binding protein C corrects an intrinsic inhomogeneity in cardiac excitation-contraction coupling. *Sci. Adv.* 1, e1400205 10.1126/sciadv.1400205 25839057 PMC4380226

[JCS210443C27] Sadayappan S. and de Tombe P. P. (2012). Cardiac myosin binding protein-C: redefining its structure and function. *Biophys. Rev.* 4, 93-106. 10.1007/s12551-012-0067-x 22707987 PMC3374655

[JCS210443C28] Seidel M., Lai F. A. and Zissimopoulos S. (2015a). Structural and functional interactions within ryanodine receptor. *Biochem. Soc. Trans.* 43, 377-383. 10.1042/BST20140292 26009179

[JCS210443C29] Seidel M., Thomas N. L., Williams A. J., Lai F. A. and Zissimopoulos S. (2015b). Dantrolene rescues aberrant N-terminus inter-subunit interactions in mutant pro-arrhythmic cardiac ryanodine receptors. *Cardiovasc. Res.* 105, 118-128. 10.1093/cvr/cvu240 25411383

[JCS210443C30] Sequeira V., Witjas-Paalberends E. R., Kuster D. W. and van der Velden J. (2014). Cardiac myosin-binding protein C: hypertrophic cardiomyopathy mutations and structure-function relationships. *Pflugers Arch.* 466, 201-206. 10.1007/s00424-013-1400-3 24240729

[JCS210443C31] Song Q., Schmidt A. G., Hahn H. S., Carr A. N., Frank B., Pater L., Gerst M., Young K., Hoit B. D., McConnell B. K. et al. (2003). Rescue of cardiomyocyte dysfunction by phospholamban ablation does not prevent ventricular failure in genetic hypertrophy. *J. Clin. Invest.* 111, 859-867. 10.1172/JCI200316738 12639992 PMC153769

[JCS210443C32] Spezzacatene A., Sinagra G., Merlo M., Barbati G., Graw S. L., Brun F., Slavov D., Di Lenarda A., Salcedo E. E., Towbin J. A. et al. (2015). Arrhythmogenic phenotype in dilated cardiomyopathy: natural history and predictors of life-threatening arrhythmias. *J. Am. Heart Assoc.* 4, e002149 10.1161/JAHA.115.002149 26475296 PMC4845125

[JCS210443C33] Stanczyk P. J., Lai F. A. and Zissimopoulos S. (2016). Genetic and biochemical approaches for in vivo and in vitro assessment of protein oligomerization: the ryanodine receptor case study. *J. Vis. Exp.* 113, e54271 10.3791/54271 PMC5065051 27500320

[JCS210443C34] Waldmuller S., Erdmann J., Binner P., Gelbrich G., Pankuweit S., Geier C., Timmermann B., Haremza J., Perrot A., Scheer S. et al. (2011). Novel correlations between the genotype and the phenotype of hypertrophic and dilated cardiomyopathy: results from the German competence network heart failure. *Eur. J. Heart Fail* 13, 1185-1192. 10.1093/eurjhf/hfr074 21750094

[JCS210443C35] Zissimopoulos S., West D., Williams A. and Lai F. (2006). Ryanodine receptor interaction with the SNARE-associated protein snapin. *J. Cell Sci.* 119, 2386-2397. 10.1242/jcs.02936 16723744

[JCS210443C36] Zissimopoulos S., Seifan S., Maxwell C., Williams A. J. and Lai F. A. (2012). Disparities in the association of the ryanodine receptor and the FK506-binding proteins in mammalian heart. *J. Cell Sci.* 125, 1759-1769. 10.1242/jcs.098012 22328519

[JCS210443C37] Zissimopoulos S., Viero C., Seidel M., Cumbes B., White J., Cheung I., Stewart R., Jeyakumar L. H., Fleischer S., Mukherjee S. et al. (2013). N-terminus oligomerization regulates the function of cardiac ryanodine receptors. *J. Cell Sci.* 126, 5042-5051. 10.1242/jcs.133538 23943880

