# Supplementary material for: Association of cardiac myosin-binding protein-C with the ryanodine receptor channel – putative retrograde regulation?
Source: J Cell Sci. 2018 Aug 3;131(15):jcs210443. doi: 10.1242/jcs.210443 (PMC6104826; doi:10.1242/jcs.210443)
Supplement: Supplementary information [file joces-131-210443-s1.pdf]

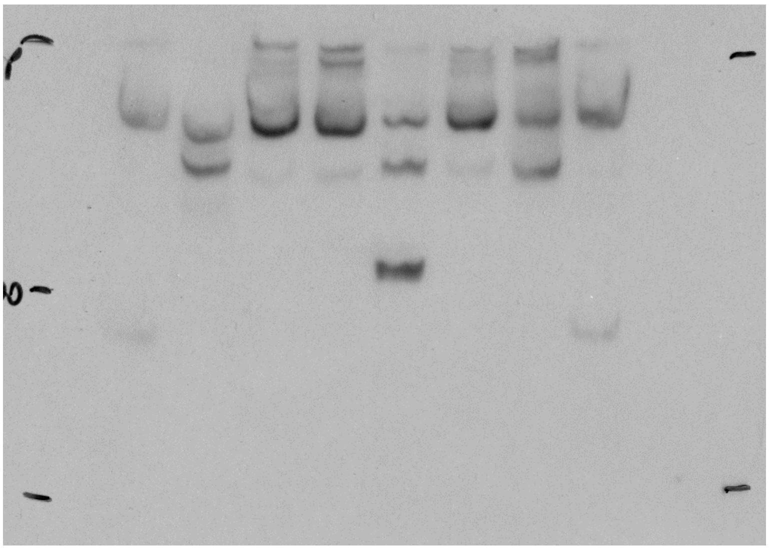

Fig. S1. Antibody P12 characterisation

1. HEK micro/ human RyR2
2. rabbit skeletal SR
3. guinea-pig CSR
4. Mouse CSR
5. Pig CSR
6. Rabiit CSR
7. Rat SCR
8. HEK micro/ human RyR2.

Journal of Cell Science • Supplementary information

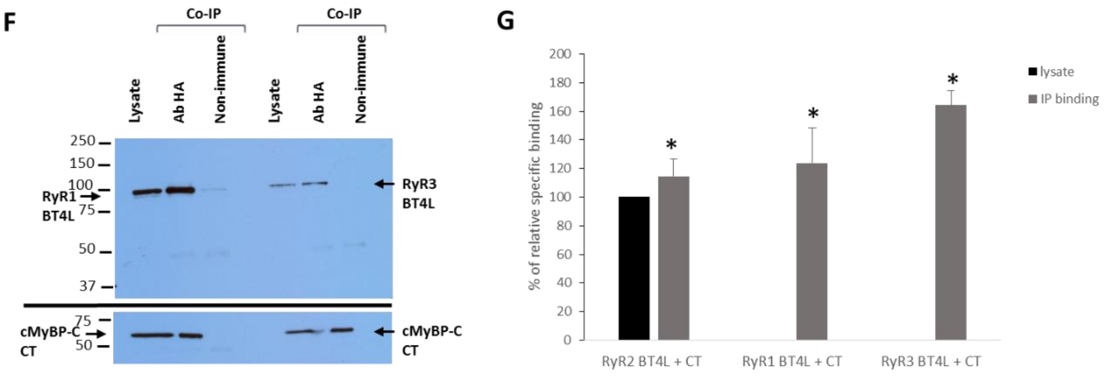

*Fig. S2. (F) Representative blot showing co-IP results of RyR1 BT4L + CT and RyR3 BT4L + CT. (top panel) WB using c-Myc Ab detecting c-Myc tagged BT construct of interest; (lower panel) corresponding WB using HA Ab detecting HA-tagged cMyBP-C CT. (G) Cumulative densitometry analysis of results shown in (F). Data in (B) and (G) is presented as relative specific binding (the specific binding subtracted of the non-specific binding) and normalised to each fragments respectable lysate input (corresponding to 1/200th of the processed sample), shown as mean ±SEM, n≥5; * statistical significance at $p<0.05$ calculated using paired, 2-tailed Student's t-test; # statistical significance at $p<0.05$ calculated using Kruskal Wallis test with Dunn's multiple comparisons.*

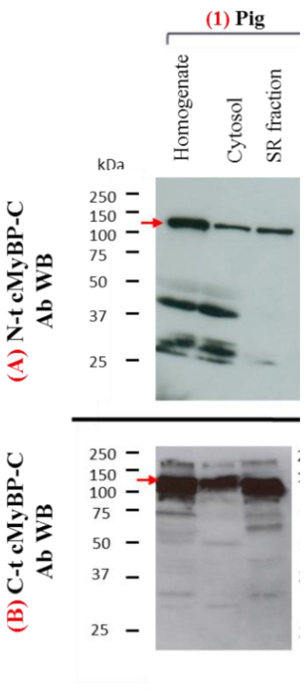

Fig. S3. Representative blots of cMyBP-C distribution in pig heart subcellular fractions. 50µg of homogenate, cytosol and SR fraction per each lane was loaded onto 10% SDS-PAGE gels and proben with either N-t or C-t cMyBP-C Ab. Band of ~140 kDa corresponding to full-length porcine cMyBP-C was detected in all fractions (red arrow).

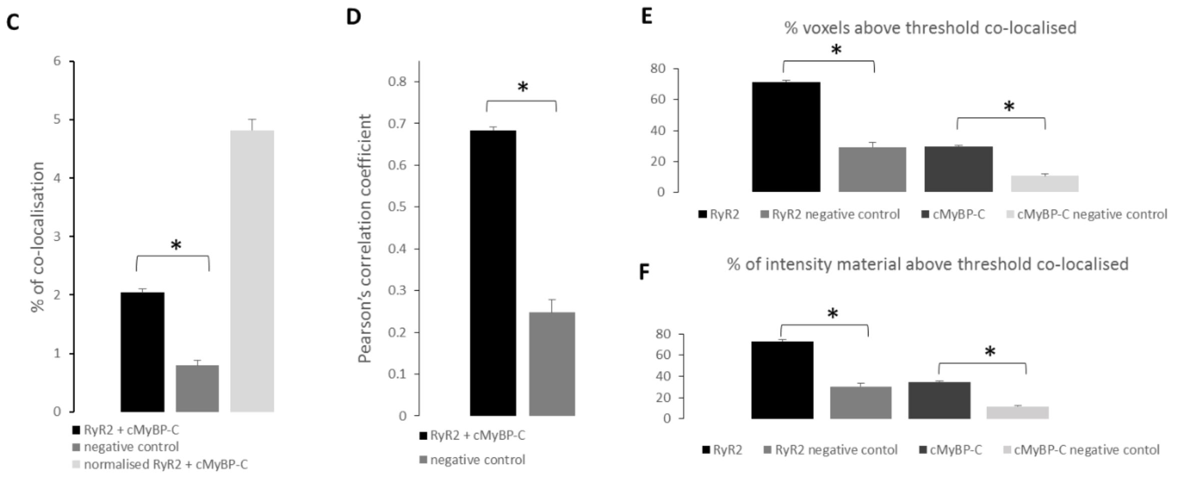

*Fig. S4. Visualisation of RyR2 and cMyBP-C partial co-localisation. (C) % of total dataset voxels co-localised, before and after normalisation to the region of interest (cell size), (D) Pearson's correlation of RyR2 and cMyBP-C inside the entire data set, (E) % of RyR2 or cMyBP-C voxels above the threshold co-localised, (F) % of RyR2 or cMyBP-C signal intensity material above the threshold co-localised. Data are shown as mean ±SEM, Samples: RyR2 + cMyBP-C n=184, negative control (representing the non-specific co-localisation results) n=40 fields of view, from four independent experiments; * statistical significance at p<0.05 calculated using unpaired, 2-tailed Student's t-test.*

Journal of Cell Science • Supplementary information

Table S1 List of primers.

Primer	Sequence	Restriction site	Length	Content	Tm (°C)	MW	Notes
**HcMyBPC.FOR.27-56**	CTC AGG ATC CCT GAG CCG GGG AAG AAG CCA	BamHI (M)	30b, 29b match HcMyBP-C	A 8, C 9, G 10, T 3	83.2	9255	F, includes 6b of the 5' UTR, 1b changed to produce a BamHI site, 4b clamp
**HcMyBPC.REV.3849-3876**	TCC CCT CGA GCC AGC CTG GTC ACT GAG G	XhoI (M)	28b, 26b match HcMyBP-C	A 4, C 11, G 8, T 5	82.8	8530	R, includes 19b of the 3' UTR, 2b changed to produce a XhoI site, 4b clamp
**HcMyBPC.REV.2611-2630**	TTA ACT CGA GGC ATG AAG GGC TGG GAG G	XhoI (A)	28b, 20b match HcMyBP-C	A 7, C 4, G 12, T 5	77.9	8762	R, 4b clamp
**HcMyBPC.FOR.2481-2508**	CGG TGA ATT CAG CTG AAC TTC GAC CTG A	EcoRI (M)	28b, 26b match HcMyBP-C	A 7, C 7, G 7, T 7	75.6	8592	F, 2b changed to produce a EcoRI site, 4b clamp
**HcMyBPC.REV.3217-3237**	A AGTC TCG AGG ACT TGG CTT GTC AAC AA	XhoI (A)	28b, 21b match HcMyBP-C	A 8, C 6, G 7, T 7	72.8	8616	R, 3b added to create XhoI site, 4b clamp

Journal of Cell Science • Supplementary information

**HcMyBPC.FOR.2925-2949**	CAG GGG ATC CTG CAA CGG CCA CGG C	BamHI (M)	25b, 24 match HcMyBP-C	A 5, C 9, G 9, T 2	84.2	7681	F, 1b changed to produce BamHI site, 4b clamp
**HcMyBPC-FOR.2331 -2360**	AGG GGG ATC CAC GTG CCA GAC GCA CTT GCG	BamHI (M)	30b, 27 match HcMyBP-C	A 7, C 10, G10, T 3	88.1	9247	F, 3b changed to produce BamHI site, 4b clamp
**HcMyBPC.REV.2931-2958**	GCA GCT CGA GCC GTG GCC GTT GCA GGA T	XhoI (M)	28b, 25 match HcMyBP-C	A 4, C 8, G 11, T 5	85.6	8650	R, 2b changed to create XhoI site, 4b clamp
**HcMyBP-.FOR.2625-56**	ATG CGG ATC CGT CCC CCC AGC GAA CCC ACC CA	BamHI (M)	32b, 29 match HcMyBP-C	A 7, C 16, G 6, T 3	90.5	9649	F, 3b changed to produce BamHI site, 4b clamp
**HcMyBP-.REV.3519-3488**	GG TCT CTA GAT AAA GAC GGG CTC CTT GTT GTT	XbaI(M)	32b, 29 match HcMyBP-C	A 6, C 6, G 11, T 9	75.5	9915	R, 3b changed to create XbaI site, 4b clamp
**HcMyBP-.FOR.3204-35**	CTG GGG ATC CAG GTT GTT GAC AGG CCA AGT CC	BamHI(M)	32b, 29 match HcMyBP-C	A 7, C 8, G 10, T 7	80.9	9869	F, 3b changed to produce BamHI site, 4b clamp

Journal of Cell Science • Supplementary information

**HcMyBP-.REV.2316-47**	GG CAT CTA GAT GAC CTT GAC TGT GAG GTT GAC	XbaI(M)	32b, 29 match HcMyBP-C	A 7, C 6, G 10, T 9	74.5	9899	R, 3b changed to produce XbaI site, 4b clamp
**HcMyBP-.REV.825-864**	C TCC CTC GAG **C**TA GCG GCG GAA GGC TGA TAG GAG GTC CAG	XhoI (M)	40b, 30 match HcMyBP-C	A 8, C 11, G 15, T 6	87.2	10159	R, 4b clamp, stop codon added
**HcMyBP-.FOR.792-824**	CTC AGG ATC CAC GAG GCC ATG GGC ACC GGA GAC	BamHI (M)	33b, 30 match HcMyBP-C	A 8, C 11, G 11, T 3	87	12388	F, 3b changed to produce BamHI site, 4b clamp
**mCherry.FOR.1-27**	CGG CAA GCT T**C**C ACC ATG GTG AGC AAG GGC GAG GAG GAT AAC	HindIII (A)	42b, 31 match mCherry	A 12, C 10, G 15, T 5	88.6	13047	F, 4b clamp.
**mCherry.REV.689-708**	T GGC GCT AGC CTT GTA CAG CTC GTC CAT GC	NheI (M)	30b, 22 match mCherry	A 4, C10, G 8, T 8	81.2	9150	R, 3b changed to produce NheI site

Notes: restriction site underlined, (M) modified residue shaded in yellow, (A) added residues highlighted in blue, (F) forward, (R) reverse.
